# A three-dimensional model of error and safety in surgical health care microsystems. Rationale, development and initial testing

**DOI:** 10.1186/1471-2482-11-23

**Published:** 2011-09-05

**Authors:** Peter McCulloch, Ken Catchpole

**Affiliations:** 1 Quality, Reliability, Safety and Teamwork Unit, Nuffield Department of Surgical Sciences, University of Oxford, Level 6 John Radcliffe Hospital, Headley Way, Oxford OX3 9DU UK

**Keywords:** Patient safety, surgery, medical error, theory, system, culture

## Abstract

**Background:**

Research estimates of inadvertent harm to patients undergoing modern healthcare demonstrate a serious problem. Much attention has been paid to analysis of the causes of error and harm, but researchers have typically focussed either on human interaction and communication or on systems design, without fully considering the other components. Existing models for analysing harm are principally derived from theory and the analysis of individual incidents, and their practical value is often limited by the assumption that identifying causal factors automatically suggests solutions. We suggest that new models based on observation are required to help analyse healthcare safety problems and evaluate proposed solutions. We propose such a model which is directed at "microsystem" level (Ward and operating theatre), and which frames problems and solutions within three dimensions.

**Methods:**

We have developed a new, simple, model of safety in healthcare systems, based on analysis of real problems seen in surgical systems, in which influences on risk at the "microsystem" level are described in terms of only 3 dimensions - technology, system and culture. We used definitions of these terms which are similar or identical to those used elsewhere in the safety literature, and utilised a set of formal empirical and deductive processes to derive the model. The "3D" model assumes that new risks arise in an unpredictable stochastic manner, and that the three defined dimensions are interactive, in an unconstrained fashion. We illustrated testing of the model, using analysis of a small number of incidents in a surgical environment for which we had detailed prospective observational data.

**Results:**

The model appeared to provide useful explanation and categorisation of real events. We made predictions based on the model, which are experimentally verifiable, and propose further work to test and refine it.

**Conclusion:**

We suggest that, if calibrated by application to a large incident dataset, the 3D model could form the basis for a quantitative statistical method for estimating risk at microsystem levels in many acute healthcare settings.

## Background

"Essentially, all models are wrong, but some are useful"

George AP Box

### Safety remains a major problem

It is only in the last 2 decades that it has become acceptable to admit that modern healthcare causes harm to patients. Early writers who drew attention to this, like Ivan Ilich[1], were dismissed as dystopian, whilst others, who raised the problems of healthcare harm after experiencing personal tragedy, were clearly not neutral observers. The medical and nursing professions have had difficulty in looking squarely at this issue, since our professional cultures contain a fundamental assumption of absolute commitment to selfless service, and the most demanding standards of performance. This mindset made it difficult to acknowledge the problem of patient harm, but eventually careful retrospective analysis in the USA showed convincing evidence of harm caused by treatment in around 3% of patients[2,3]. Studies from other advanced health care systems now suggest that this is probably a considerable under-estimate[4-6]. Regulatory authorities have subsequently commissioned reports[7,8] which have identified inadvertent harm as a major healthcare problem, and called for urgent reform. In this paper we offer an early presentation of a conceptual model that seeks to link the high frequency of incidents and near misses to individual contributing events attributable to causes within care Microsystems. We discuss the possibility that this could be developed into a statistically explorable model for error analysis, and prediction of intervention effects.

Like early treatments for infections based on inadequate theoretical understanding[9], our early efforts to reduce healthcare-related harm have often had limited success[10]. Analysis of the problems encountered during such attempts reveals complex interactions between the objects of interventions - for example work systems - and other aspects of the organisation, such as staff culture[11]. Thus, understanding and predicting complex systemic interactions is important for the design of effective interventions. A variety of theoretical models and frameworks have been proposed to help classify, understand and analyse the causes of error and harm in healthcare. The Reason "Swiss Cheese" model[12] has had an important role in illustrating the complexity of error, and providing an easily understandable conceptual approach. Amalberti[13] and Hollnagel[14] offer other examples of theoretically plausible and partially validated socio-technical conceptual models, although the latter has not been specifically tested in health care. Analysis frameworks for individual events or collections of incidents, such as the London protocol[15] aim to describe the influence of the entire range of factors which may contribute to errors. Carayon and Smith propose a 5 dimensional model of influences, which includes Environment and Organisation, Tasks, People and Tools[16]. The WHO classification system is also based on a single incident classification system, with explicitly described categories[17]. Unfortunately, these models tend to be conceptual and communicative rather than statistically testable or predictive. They are mainly based either on human factors theory or analysis of small numbers of unusual incidents with serious consequences. Because they attempt to explain incidents comprehensively, they inevitably include concepts and influences which are either very difficult to define and evaluate (such as "management ethos") or very difficult to change in real health care practice (such as Organisation or People). This can easily result in unclear conclusions or infeasible recommendations. A model which would allow analysis of problems at the microsystem level, and which avoided or compartmentalised such unmanageable issues would be potentially very useful. Furthermore, the emphasis on understanding individual events means that models designed and deployed to describe major "accidents" can provide over-specific analyses, and do not always lend themselves well to multi-incident analysis. This may be sufficient for safety in some domains (especially transport, where one "error" can have an impact on hundreds of passengers), but the risk with a root-cause analysis model is of providing event-specific solutions rather than identifying common causes. Observational research work has broadly identified the recurring types and sources of everyday incidents that may eventually contribute to more serious incidents[18-21], but such frequent events cannot be addressed using the complex analyses generally required by theoretical models. Arguably the most obvious failure of existing models is to predict the effects of different interventions. We believe that there is therefore a need for a practical healthcare safety model which clarifies the relative importance of, and interactions between, the factors affecting patient safety at the "microsystem" level where patient care is actually delivered, and which can be used to understand both individual and recurring events.

## Methods

To develop our model, we used the following 6 propositions:

We should be able to define any concept we use in the model, and the definitions should be consistent with current usage.

• We should accept concepts or influences into the model only if their relationship with harm has been verified in well designed experiments, or high-quality formal observational studies.

• We should not accept any influences which can be completely described as a composite of other more fundamental influences

• We should use only as many influences as are required to explain observed occurrences (Occam's razor)

• We should test the model repeatedly against observed reality to see if it provides a satisfactory explanation of what occurred.

• We should refine the model by using it to make predictions and comparing these with future real events.

Using these principles, we contend that *System, Culture *and *Technology *represent three concepts which have considerable experimental support as influences on error and safety[20,22-27], and which between them can encompass most of the manageable influences active at the microsystem level. The concept of healthcare workplace safety *culture *has been defined in detail, and measures for it have been developed[28-30]. The importance of *systems *analysis has been repeatedly emphasised in reviews of the scientific evidence on healthcare safety31,32]. *Technology *can enhance safety, for example via decision support[33] and "smart" technology to improve drug administration[34], or increase dangers. All three terms have been previously defined in various ways, but in order to avoid confusion we have chosen here to use the definitions which appear best validated and most widely accepted in the current literature.

*Work systems*are the ways in which work is conducted, including the formal organisation of tasks and responsibilities as well as the precise manner of achieving them. It therefore incorporates the manner of information handover, standard procedures for preparing and using equipment, and the pathways by which patients move through the system. This definition encompasses both Donabedian's dimensions of *process *and of *structure*[35].

*Workplace culture*has been defined in various ways[28-30]. Here we mean the values, attitudes and assumptions which guide and underpin staff relationships and communication. This includes local notions of hierarchy, loyalty and professionalism, and perceptions of the work environment, patients, other staff groups and management.

*Technology*is the equipment used to carry out the tasks assigned. It includes both complex and simple instruments and tools, but also drugs, medication and information technology.

### Summary of model

**Figure 1:**
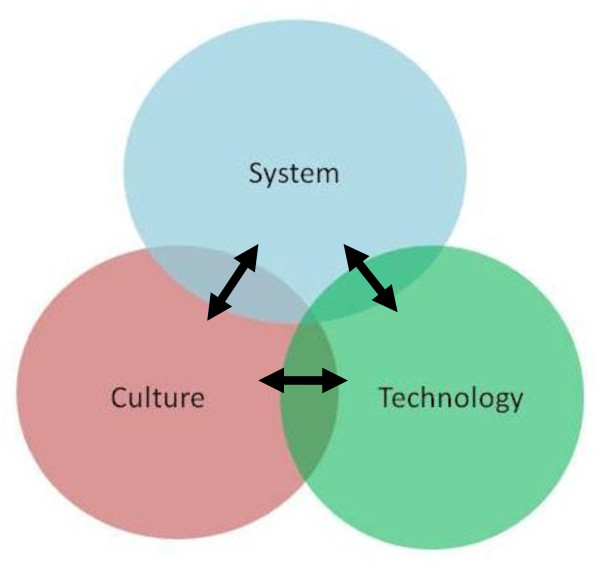
**The 3D model of influences on patient safety and risk**. Interactions between dimensions may be negative or positive, uni or bi directional, and may take multiple forms.

We propose a model of healthcare safety in which threats to patient safety at the ward, unit or theatre level can be analysed in terms of the 3 dimensions of *System*, *Culture *and *Technology*. Based on the conceptual model proposed by Reason[12], we assume that serious threats to safety result from a *stochastic *process, whereby chance combinations of small errors or defects in any of the 3 dimensions "snowball" or link up unpredictably to cause major accidents or harm. The dimensions are however *interdependent*, i.e. changes in one dimension have important effects on other dimensions, which may enhance or decrease safety risks, or modify them in a variety of other ways (see Figure 1). For example a culture defect might allow a systems error to persist, which would have been quickly rectified in a more appropriate culture (see example, below). Unlike the Reason model, therefore, this 3 dimensional model recognises the fact that safety defects interact with each other more dynamically than by "lining up", and that this interactivity is very important in understanding and tackling them. Conversely, the model provides a framework for describing the ways in which systems successfully prevent error, by arranging mutually supportive interactions between dimensions to promote resilience.

### Advantages of this model

This simple model has some useful properties for a theoretical framework for research on healthcare safety at the micro level:

1. It can be applied to all internal threats to safety at microsystem level that we have so far identified.

2. It permits a plausible description of the way in which flawed systems, flawed attitudes/culture and inappropriate technology can form a mutually supportive vicious circle.

3. Conversely, it explains the ineffectiveness of interventions focussed on only one dimension: This leaves two dimensions untouched, and interactivity means that changes in these other dimensions, either in response to the intervention or unconnected, can undermine effectiveness.

4. It predicts that reacting to individual events by legislating for the exact circumstances which caused them is futile, because novel stochastic combinations of small imperfections will inevitably emerge.

5. It therefore predicts that reducing the overall level of identifiable small imperfections in all three dimensions (culture, system and technology) will reduce the incidence of harm incidents. This is open to experimental verification.

6. It predicts that, for maximal effectiveness, interventions should address all three dimensions, attempting to achieve synergy in a manner similar to that used in combined chemotherapy, where drugs target different metabolic mechanisms. Again, this hypothesis can be tested.

In order to carry out some preliminary testing of whether these three concepts are sufficient to describe the complexity of real situations, we analysed some real cases observed during our studies in surgery, in which harm could have or did occur[11,20]. We used a database of recorded safety incidents recorded during an observational study of safety on an emergency surgery unit[20]. We analysed the influences contributing to a random sample of 12 readmissions and complications after discharge, using data available in study records. The attribution of causation presented was taken from study records, edited only where necessary to clarify how incidents could be completely described using the three concepts of culture, system and technology.

## Results

Of a sample of 12 readmissions and complications after discharge to an emergency surgery unit we analysed, we found that the underlying causes could immediately be described in terms of 1 (4/12) 2 (3/12) or all 3 (2/12) of these dimensions. The other incidents required additional information from case notes to analyse them, but when this was obtained there were none which required the use of other dimensions to explain them fully. In this particular sample we found that the influences were involved in a total of 7/12 (system), 7/12 (culture) and 2/12 (technology) incidents respectively. Interactions of two influences were most commonly between culture and system. What was most valuable was the way in which the self-reinforcing adverse interactions between factors were highlighted by this manner of analysis (see below and Table 1)

**Table 1:** 3D analysis of some example incidents in emergency general surgery

Case	Description of Problem	Analysis of Contributory Factors	Classification	Commentary
1	Long term Crohn's disease: Emergency stricture resection for obstruction. ARDS postoperatively	Overhydration, no antibiotics: 2y to deficient protocols, supervision, training.	Culture +System failures	Culture accepted system where undertrained juniors did their best without adequate supervision - as no alternative appeared possible.

2	91 yr old with apparently strangulated hernia, but negative exploration, followed by MI	Inappropriately rapid decision to operate without investigation	Culture failure	Radiology and senior consultation available, but not accessed due to "macho" culture. Trainee's perception of their role emphasised self- reliance and decisiveness, and characterised use of expensive investigations and discussion with seniors as indicators of lack of experience and competence

3	Possible intestinal obstruction; significant arm soft tissue injury from CT contrast extravasation	Lack of protocol and experience, plus equipment which hinders immediate detection and cessation of injection	Culture, System and Technology failure	System could have been devised to defend against obvious risks of technology but culture did not demand it. More detailed and explicit protocol incorporating checks of intravenous access quality before and during infusion needed: however culture resistant to close adherence to standardised protocols, which is regarded as interfering with professional freedom

4	Second laparotomy for ileus after initial division of adhesions	Overhydration, late withdrawal of epidural: 2y to lack of protocols	System + Culture failures	Lack of protocols, Failure of culture to insist on closer surveillance. Inadequate handover of information at shift changes. Excessive workload at times, putting pressure on junior doctors' memory and stamina.

5	Schizophrenic with missed caecal volvulus; anastomotic leak, 2^nd ^laparotomy, ITU	Failure to perform appropriately timely investigations or postop surveillance in difficult patient	Culture failure	System malfunctioned due to cultural acceptance of suboptimal care for "difficult" patients. Blood tests and observations omitted to avoid challenging situations

6	Pneumonia and resp. failure after long laparotomy	Inadequate analgesia from failed epidural, then oversedation from incorrectly prescribed PCA	System & Technology failures	Inadequate access to prompt expert pain management; PCA pump easy to misuse. No clear detailed protocol for epidural or PCA care. De-skilling of junior medical and nursing staff by reliance on specialist pain team who were not always promptly available. Juniors reluctant either to make decision to change analgesic strategy or to ask for senior guidance.

An illustrative example of the method taken from previous observation in our earlier work[11] is as follows:

*The CO2 supply runs out at a critical moment in a laparoscopic operation, and harm occurs because there is a significant delay in re-establishing pneumoperitoneum, caused by (a) delay in finding a new bottle of CO2 (b) inexperienced staff failing to correctly connect the new bottle (c) a maintenance failure making the insufflators unpredictable and (d) lack of understanding by the surgeon of the controls on this particular model of insufflator*.

*Here several influences have interacted. There are technology problems (multiple insufflators types, poor maintenance) training defects (in both nursing and surgical personnel) and work system faults (CO2 bottles not readily available: training profile of staff inadequate for procedure). According to our understanding of underlying causes, the key training defects are largely a culture problem, caused by a "carry on regardless" attitude and the belief that professionals do not need externally regulated training. The easily foreseeable risk of this type of incident is commonly "traded off" against reducing work-rate, and most practising surgeons would recognise this example as typical of the dangers of everyday practice. Our culture permits a work process where systems analysis would identify multiple avoidable high risk areas, including working with equipment well known to be unreliable, or for which the team has inadequate training*.

## Discussion

The model closest to our suggestion in the current literature is the SEIPS model developed by Carayon and Smith. They propose a 5 dimensional model for organisational error, which includes Environment and Organisation, Tasks, People and Tools[16]. Our model collapses Tasks and part of Organisation into the System dimension, whilst People and parts of Organisation and Environment are incorporated into Culture, with Technology approximating to Tools. We would suggest that our model allows equally precise analysis in most cases, with a more parsimonious approach which directs attention towards correctable problems in the health care organisation. In most real research and practice applications of safety science, the infrastructure and the personnel are not easily modified, but are all too easily blamed in post-hoc incident analysis; therefore little is lost and a good deal may be gained by excluding them as remediable factors in our model. The 3 dimensions have a direct bearing on all activity at microsystem level which is obvious to the observer, and focussing on them therefore concentrates analysis on the tangible and remediable. As Reason points out[36], safety analysis needs to be constrained by what is remediable within an organisation, and causation becomes progressively more difficult to determine as one moves from the active error towards concepts such as "management ethos". A methodology which can clearly define the influences evident at "microsystem" level has obvious applicability which can easily be lost in more complex frameworks such as SEIPS or Vincent's London protocol [[12], as Molloy et al point out[37]. Where higher-level influences are important, this will normally be evident from the conclusions of the 3D analysis. In the extended example, the tolerance of training and maintenance defects begs obvious questions about priorities at higher levels in the organisation.

### Limitations

Although we argue for a data-driven model, we concede that this early proposal is derived from literature analysis, reasoning and limited testing, and therefore requires considerable further validation work to demonstrate its utility relative to existing frameworks. The analysis of our cases might have had greater face validity if we had asked an objective external observer to carry it out, although this would have required detailed explanation of the concepts followed by a training process.

Because this model is focussed on analysis and prediction at the microsystem level, it does not directly deal with the higher level influences which may affect this level. For example, selection and training of personnel, staffing levels and management policies on issues such as discipline and risk management are issues which can have major effects on safety at the microsystem level. We would argue, and so far our experience supports this, that these higher level influences necessarily act via their effects on the three dimensions we focus on. Therefore higher level influences will not necessarily be missed by using the model: rather their importance will emerge from detailed analysis of the nature of the specific culture, technology or system defects identified in any specific case.

## Conclusions

We believe this theoretical model may be useful in several ways: to classify and understand threats to safety: to develop tools for analysing incidents: and to generate hypotheses about potential safety interventions, for experimental testing. We believe there is potential for developing a quantifiable measure of system risk by applying statistical analysis to real outcomes data, and modelling the assignment of different values to the risk elements in each dimension. As the basis for an audit approach, this could give an institution a clearer understanding of its' greatest threats to safety. Allied to an FMEA-type prospective survey of risk, it could enhance understanding of "latent conditions" and guide prophylactic action. We propose to develop some of these lines of enquiry in future studies, and would welcome feedback and discussion from others interested in this field of work.

There is a need for a simple but comprehensive model to explain errors and harm in surgical care and to evaluate preventative interventions at the level of direct patient care. We propose a model which uses only three dimensions to classify problems and solutions, uses standard definitions of terms, and is supported by limited testing on observation of real instances. The model leads to a testable hypothesis on the effectiveness of safety solutions and has a number of properties which suggest it may be useful in healthcare quality improvement practice.

## Abbreviations

SEIPS stands for Systems Engineering for Improving Patient Safety. This is one of several existing models for analysing safety incidents in healthcare. FMEA stands for Failure Modes and Effects Analysis. This is a well recognised industrial technique for evaluating risk in complex systems and estimating the product of probability and potential impact to prioritise the most important sources of possible error.

## Competing interests

The authors declare that they have no competing interests.

## Authors' contributions

PM wrote the first and final drafts and had the original idea. KC helped to develop the idea in discussions over a period of months, and provided expertise and advice based on a professional background in Human Factors. Both authors read and approved the final version of the manuscript.

## Pre-publication history

The pre-publication history for this paper can be accessed here:

http://www.biomedcentral.com/1471-2482/11/23/prepub
